# John W. Clippinger

**Published:** 1901-04

**Authors:** 


John W. Clippinger, a student in the Freshman class of the medical department of the University of Buffalo, died at Buffalo General Hospital, Thursday, March 21, 1901, from endocarditis. Funeral services were held next day, attended by the faculty and students, after which the remains were sent to Pipestone, Mich., the home of the deceased.

